# Changing Colorectal Cancer Trends in Asians: Epidemiology and Risk Factors

**DOI:** 10.3389/or.2023.10576

**Published:** 2023-05-22

**Authors:** Carissa Ikka Pardamean, Digdo Sudigyo, Arif Budiarto, Bharuno Mahesworo, Alam Ahmad Hidayat, James W. Baurley, Bens Pardamean

**Affiliations:** Bioinformatics and Data Science Research Center, Bina Nusantara University, Jakarta, Indonesia

**Keywords:** epidemiology, screening, colorectal cancer, Asian population, risk factor

## Abstract

Once an infrequent disease in parts of Asia, the rate of colorectal cancer in recent decades appears to be steadily increasing. Colorectal cancer represents one of the most important causes of cancer mortality worldwide, including in many regions in Asia. Rapid changes in socioeconomic and lifestyle habits have been attributed to the notable increase in the incidence of colorectal cancers in many Asian countries. Through published data from the International Agency for Cancer Research (IARC), we utilized available continuous data to determine which Asian nations had a rise in colorectal cancer rates. We found that East and South East Asian countries had a significant rise in colorectal cancer rates. Subsequently, we summarized here the known genetics and environmental risk factors for colorectal cancer among populations in this region as well as approaches to screening and early detection that have been considered across various countries in the region.

## Introduction

Asia is home to approximately 61% of the world’s population, representing the most densely populated continent in the world [1]. Colorectal cancer (CRC) is most commonly diagnosed in Australia and New Zealand, followed by Europe and North America. The highest rates of death from CRC are reported in Central Eastern Europe. In contrast, the regions with the lowest incidence of CRC are South Asia and Africa, which also report the lowest mortality rates. However, these areas have the highest ratios of mortality-to-incidence [2]. In particular, Asia has been facing significant changes in the epidemiology of several gastroenterological diseases, including colorectal cancer [3]. The rapid changes in socioeconomic and lifestyle habits, including sedentary lifestyles, obesity, tobacco use, as well as consumption of spicy foods, alcohol, and meat are considered to contribute to cancer incidence and mortality trends in many Asian countries [4–6]. Such changes pose challenging health-care-related issues regarding prevention, early detection, and treatment of gastrointestinal malignancies in developing countries within the Asian continent. In this review, we summarized and discussed the changes in the epidemiology of colorectal cancer that has occurred over the past few decades, focusing specifically on Asians and possible strategies for early detection and treatment.

## Descriptive Epidemiology

**FIGURE 1:**
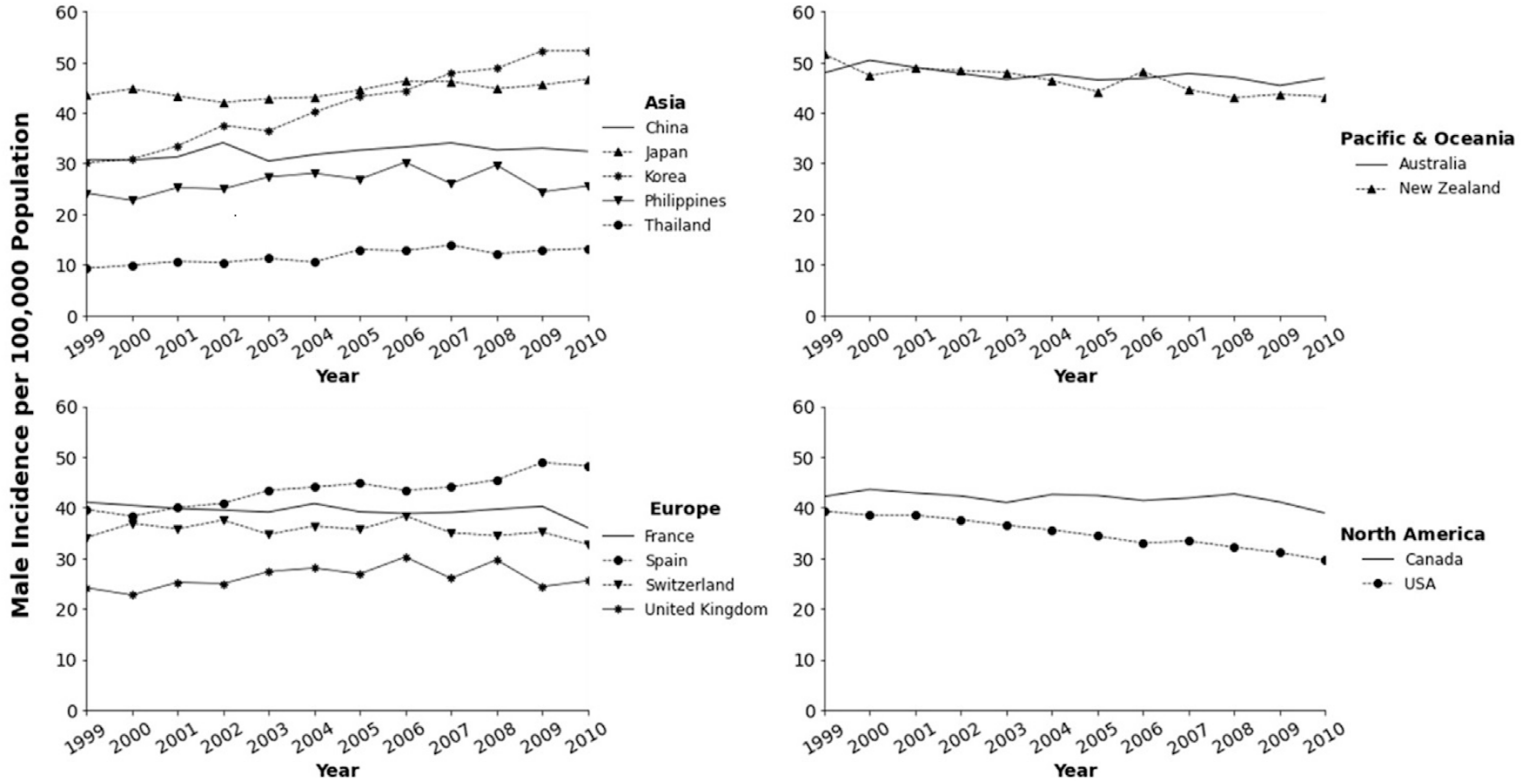
Trends in colorectal cancer incidence among men in Asia-Pacific, North America, and Europe from the cancer incidence in five continents (CI5) database.

**FIGURE 2:**
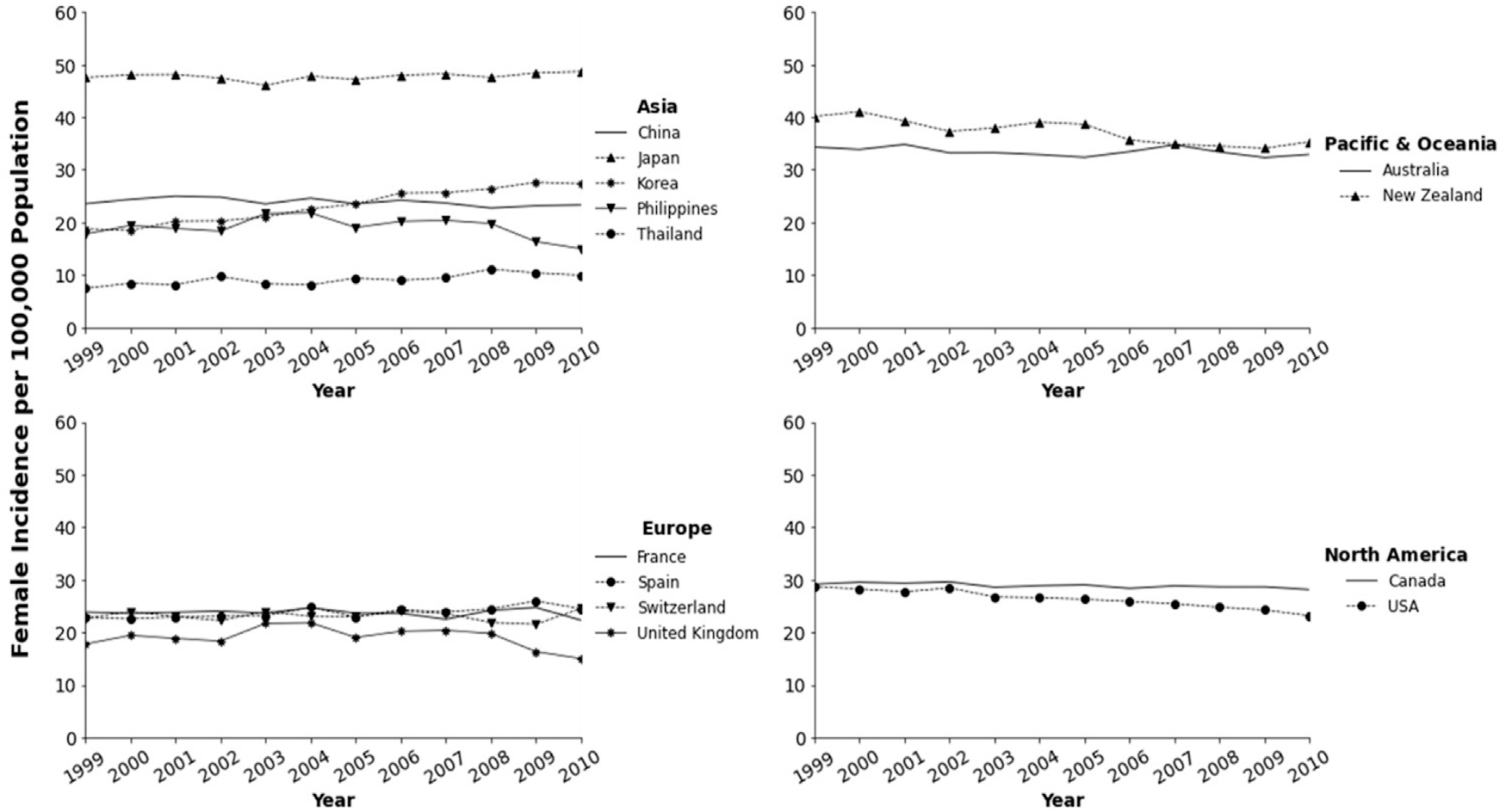
Trends in colorectal cancer incidence among women in Asia-Pacific, North America, and Europe from the cancer incidence in five continents (CI5) database.

**FIGURE 3:**
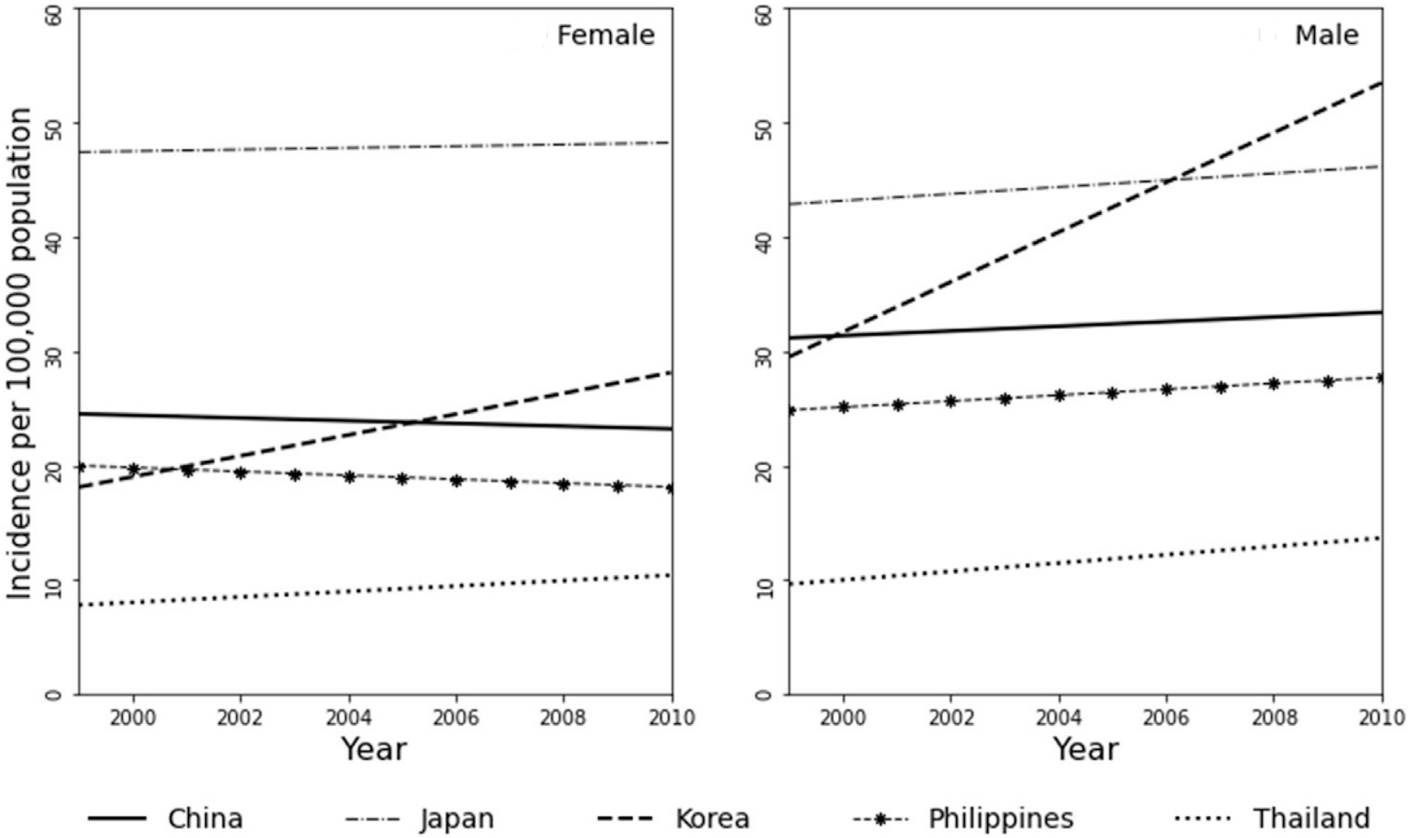
Simple regression lines based on 1999–2010 Colorectal Cancer incidence (age-standardized) rates in select Asian countries using data from the cancer incidence in five continents (CI5) with the female population in the left panel and male population on the right panel.

In parts of North America, Australia, and Europe, colorectal cancer ranks as the second or third most common cancer type; it is also the second or third leading cause of cancer-related deaths in both men and women combined. Several studies have referred to colorectal cancer as a “Western” disease [7–9]. A few decades ago, studies have documented the lower prevalence of colorectal cancer among several Asian countries as well as among Asian immigrants to the United States [10]. More recently, incidence rates in various developing countries appear to be notably high, paralleling those in developed countries such as Japan and Korea. Data from Cancer Incidence in Five Continents (CI5) published by the IARC shows an increase in rates for various Asian countries and decreasing rates for some countries in North America, Pacific/Oceania, and Europe (Figures 1, 2; Table 1). Overall the best fitting line for the age-standardized incidence rates from 1993 to 2010 in various countries of Asians is steadily increasing with some variability by country and possibly sex (Figure 3).

**TABLE 1:** Colorectal cancer incidence in Asia-Pacific, North America, and Europe among women and men using data from cancer incidence in five continents (CI5).

ASR—Incidence (per 100K population)															
	1999	2000	2001	2002	2003	2004	2005	2006	2007	2008	2009	2010	% change 1999–2010	B	SE
Female															
Asia															
China	23.52	24.35	24.98	24.78	23.49	24.59	23.51	24.19	23.65	22.71	23.14	23.29	−0.98	−0.12	0.05
Japan	47.55	48.09	48.14	47.47	46.06	47.81	47.18	48.02	48.26	47.56	48.44	48.71	2.44	0.07	0.056
Philippines	17.77	19.42	18.85	18.34	21.69	21.82	19.04	20.19	20.39	19.8	16.34	15	15.59	−0.17	0.168
Korea	18.78	18.47	20.22	20.29	21.04	22.57	23.46	25.54	25.64	26.37	27.61	27.33	45.53	0.91	0.05
Thailand	7.44	8.42	8.14	9.72	8.34	8.11	9.4	8.97	9.44	11.11	10.4	9.94	33.6	0.24	0.057
Oceania/Pacific															
Australia	34.33	33.86	34.82	33.22	33.25	32.88	32.37	33.45	34.74	33.38	32.32	32.92	4.11	−0.11	0.64
New Zealand	40.14	41.05	39.3	37.32	37.95	39.06	38.67	35.65	34.86	34.5	34.09	35.32	12.01	−0.59	0.093
North America															
Canada	29.13	29.5	29.31	29.58	28.56	28.85	29.01	28.34	28.83	28.63	28.63	28.11	3.5	−0.1	0.025
United States	28.69	28.22	27.69	28.44	26.72	26.57	26.27	25.85	25.41	24.74	24.23	23.14	19.34	−0.47	0.034
Europe															
France	23.83	23.59	23.79	23.99	23.63	24.65	23.63	23.59	22.48	24.16	24.69	22.31	6.38	−0.05	0.061
Spain	22.86	22.57	22.92	23.1	23.12	24.74	22.93	24.28	23.83	24.43	25.94	24.47	7.04	0.22	0.055
Switzerland	22.62	23.94	22.97	22.24	23.94	23.04	22.96	23.99	23.56	21.87	21.54	24.49	8.27	−0.01	0.081
United Kingdom	24.03	23.74	23.16	22.69	22.57	23.22	23.67	23.83	24.61	24.85	25.3	25.42	5.78	0.19	0.057
Male															
Asia															
China	30.73	30.67	31.31	34.11	30.48	31.75	32.64	33.28	34.07	32.69	33.01	32.39	5.4	0.2	0.091
Japan	43.46	44.74	43.31	42.08	42.81	43.11	44.53	46.32	46.21	44.79	45.55	46.65	7.34	0.3	0.093
Philippines	24.15	22.72	25.24	24.95	27.35	28.06	26.89	30.25	26.01	29.68	24.4	25.54	5.76	0.26	0.18
Korea	30.14	30.89	33.45	37.54	36.43	40.17	43.23	44.4	47.87	48.79	52.25	52.27	73.42	2.17	0.086
Thailand	9.29	9.89	10.65	10.44	11.24	10.51	12.99	12.76	13.88	12.15	12.82	13.2	42.09	0.36	0.062
Oceania/Pacific															
Australia	47.9	50.4	49	47.8	46.6	47.6	46.5	46.8	47.8	47	45.4	46.9	−2.09	−0.25	0.082
New Zealand	51.6	47.4	48.8	48.4	48	46.4	44.2	48.2	44.6	43	43.7	43.2	−16.28	−0.65	0.117
North America															
Canada	42.2	43.6	42.9	42.3	41	42.6	42.4	41.4	41.9	42.7	41.1	38.9	−7.82	−0.02	0.082
United States	39.3	38.5	38.5	37.6	36.5	35.6	34.4	33	33.4	32.2	31.1	29.6	−24.68	−0.87	0.039
Europe															
France	41.06	40.44	39.82	39.5	39.13	40.8	39.17	38.83	39.04	39.68	40.25	36.04	−12.23	−0.22	0.09
Spain	39.62	38.32	40	40.85	43.41	44.06	44.81	43.4	44.09	45.49	48.89	48.27	21.83	0.86	0.098
Switzerland	34.07	36.9	35.8	37.54	34.73	36.37	35.66	38.37	35.03	34.47	35.17	32.77	−3.82	−0.15	0.129
United Kingdom	36.32	36.73	35.24	34.92	35.25	35.93	35.96	36.51	36.41	37.95	38.11	38.36	5.62	0.23	0.07

Based on the most recent incidence data reported in GLOBOCAN 2020 from the International Agency for Research on Cancer [11], Japan has the highest estimate based on the age-standardized incidence rate (ASR) category (men: 47.3 per 100,000 and women: 23.5 per 100,000); the ASR in Brunei (men: 42.2 per 100,000 and women: 27.7 per 100,000) and Singapore (men: 38.6 per 100,000 and women: 27.4 per 100,000) are similarly high (Table 1). By comparison, the ASR in the United States, Australia, and the United Kingdom were 24.7, 30.4, and 31.5 per 100,000, respectively in men and 19.8, 23.6, and 23.6 per 100,000 in females, suggesting that rates in Asian countries may be approaching the rates of westernized countries. Several Asia-Pacific initiatives have also reported this rising incidence observed during the mid-to-late 2000s [3, 12]. Currently, Asia contributes to 49% of the total number of new cancer cases in the world, nearly half of which are found in China [11, 13]. The trend of colorectal cancer in China indicates a significant increase in incidence in both genders [14]. Studies of ethnically diverse countries have found that Chinese ancestry populations have higher incidence rates than Malay and Indian ancestry populations [15–17]. With these increasing age-standardized rates, colorectal cancer is now in the top three most common cancers in many Asian countries [18].

Overall, the ASR appears to be consistently higher in men compared to women (ratio of men to women in Brunei = 1.52; Japan = 1.55; South Korea = 1.69; Singapore = 1.41) paralleling those found in the United States (ratio = 1.25), Australia (ratio = 1.29), and Europe (ratio = 1.45). Furthermore, other studies in Asians reported preliminary comparisons between left-sided and right-sided colorectal cancer [19, 20]. Some suggest an increasing prevalence of right-sided colon cancer, especially in women and the elderly [20–22], which trends have also been observed in other parts of the world [23, 24].

Similarly, the estimated cancer deaths in Asia constitute 58% of the global population in 2020 [11]. As observed in Table 2, mortality rates in Japan, South Korea, and Singapore are currently similar to those of North America, Pacific/Oceania, and Europe. Temporal changes in mortality from colorectal cancer have been documented in previous studies [25] and the overall mortality in several Asian countries is expected to rise over the next two decades [13].

**TABLE 2:** Estimated crude and age-standardized rate (ASR) of colorectal cancer incidence and mortality among men and women in selected countries (Globocan 2020).

	Incidence						Mortality					
	Men			Women			Men			Women		
	Crude rate	ASR (W)	Risk	Crude rate	ASR (W)	Risk	Crude rate	ASR (W)	Risk	Crude rate	ASR (W)	Risk
Asia												
China	43.1	28.6	1.84	16.3	15.6	1.24	11.2	9.0	0.78	9.2	6.1	0.51
Japan	131.6	47.3	4.98	74.3	23.5	2.57	42.3	15.0	1.60	35.9	9.2	0.83
North Korea	27.3	22.8	3.20	26.8	18.5	2.29	15.4	15.0	1.75	15.9	10.1	1.25
South Korea	65.4	34.9	6.98	56.4	33.3	3.89	21.3	14.6	1.51	16.4	7.8	0.74
Mongolia	4.5	6.6	0.55	4.8	6.3	0.79	2.6	4.0	0.33	3.4	4.5	0.56
Brunei	40.1	42.2	3.85	14.7	17.4	1.43	10.1	14.1	1.83	6.8	8.5	0.63
Cambodia	9.3	13.7	1.27	5.3	6.6	0.78	4.6	8.2	0.97	3.9	5.0	0.58
Indonesia	15.8	16.5	1.90	9.6	10.1	1.18	8.7	10.8	1.28	6.4	6.7	0.77
Lao PDR	10.8	16.1	1.21	5.4	7.7	0.87	4.4	7.7	0.90	4.0	5.7	0.64
Malaysia	21.3	21.2	2.51	13.7	15.7	1.83	8.6	10.5	1.23	7.1	8.3	0.93
Myanmar	10.3	11.8	1.18	7.2	7.4	0.85	6.5	8.0	0.91	5.4	5.7	0.65
Philippines	17.5	23.7	1.86	8.1	11.0	1.30	5.6	9.7	1.12	4.6	6.4	0.72
Singapore	67.3	38.6	4.68	44.8	28.0	3.24	20.7	14.7	1.70	15.1	9.1	1.00
Thailand	31.4	19	1.76	14.2	10.1	1.17	11.2	9.0	1.01	8.5	6.0	0.68
Timor-Leste	6	10.1	1.98	5.2	9.1	0.88	6.6	14.0	1.48	3.8	7.1	0.64
Viet Nam	18.3	17.6	1.34	9.3	9.0	1.05	7.0	8,0	0.94	6.3	6.1	0.72
Pacific/Oceania												
Australia	47.4	30.4	5.29	61.6	32.0	3.61	19.9	10.7	1.16	16.5	7.6	0.76
New Zealand	48.3	29	4.79	64.5	33.5	3.78	30.7	16.8	1.83	28.6	13.7	1.44
North America												
United States	38.9	24.7	3.25	40.9	22.0	2.42	18.4	11.0	1.19	16.6	7.7	0.77
Canada	52.3	28.3	4.98	60.6	28.5	3.23	25.7	13.2	1.41	21.6	8.8	0.86
Europe												
France	53.8	28.8	4.32	59.3	24.9	2.81	29.1	12.9	1.34	25.1	8	0.74
Spain	72.1	38.7	5.20	54.9	24.2	2.74	37.8	17.1	1.80	25.2	8.4	0.82
Sweden	44.9	24	3.83	64.3	26.5	3.11	29.4	12.2	1.29	28.2	9.7	1.00
United Kingdom	55.4	31.5	4.22	57.0	24.4	2.73	28.2	13.0	1.38	23.5	8.7	0.85

## Risk Factors

### Genetics

Several genetic factors and lifestyle behaviors are known risk factors for colorectal cancer. Heritability estimates for colorectal cancer are 12%–35% [26]; however, high penetrant, germline mutations account for less than 5% of these cancers [27, 28]. To date, there are a total of 14 genes that are suspected to cause different subtypes of colorectal cancer, including mutations in adenomatous polyposis coli (APC) leading to a predisposition to familial adenomatous polyposis (FAP) and defects in mismatch repair genes in Lynch Syndrome [28].

Recently, genome-wide association studies have led to the identification of less-penetrant but more frequent genetic variants that contribute to colorectal cancer predisposition, further elucidating a larger proportion of the familial risk associated with the disease. Over 40 variants have been identified, highlighting the importance of several biological pathways, including the TGF-beta/BMP pathway and the mitogen-activated protein kinases (MAPK) pathway [28]. However, many of these genetic associations discovered in European-ancestry populations exhibit either a weak or no association with colorectal cancer in other ancestry groups, demonstrating the necessity for studies in diverse populations worldwide [29].

The Asia Colorectal Cancer Consortium, initiated in 2009 among East Asian nations, has successfully identified novel relevant, genetic regions associated with colorectal cancer in the Asian population, as seen in Table 3. Despite these large concerted efforts, the overall number of genetic variants identified appears lower than the anticipated number to account for the estimated proportion of the familial component of the disease. This missing heritability may be in part explained by gene-environment interactions, which such studies would necessitate even larger consortium efforts across multiple ethnic/racial populations. However, these findings mainly represent the East Asian populations, while the rest of the Asian populations remain underrepresented such as South, Southeast, and West Asian countries. Only limited studies have involved these populations, including Indonesia, Malaysia, and Thailand population [33–36].

**TABLE 3:** Novel variants associated with colorectal cancer in Asian population.

Rsid	Chr	Pos	Genes	Study
rs647161	5	134499092	—	[30]
rs10774214	12	4368352	Near CCND2	[30]
rs2423279	20	7812350	—	[30]
rs11196172	10	114726843	Near TCF7L2	[31]
rs704017	10	80819132	Near ZM1Z1-AS1	[31]
rs174537	11	61552680	Near MYRF	[31]
rs1535	11	61597972	Near FADS2	[31]
rs174550	11	61571478	Near FADS1	[31]
rs4246215	11	61564299	Near FEN1	[31]
rs10849432	12	6385727	Near CD9	[31]
rs12603526	17	800593	Near TGBF1	[31]
rs2241714	19	41869392	Near B9D2	[31]
rs201395236	1	245181421	EFCAB2	[32]
rs7542665	1	62673037	L1TD1	[32]
rs7606562	2	48686695	PPP1R21	[32]
rs113569514	3	133748789	Near SLCO2A1	[32]
rs12659017	5	125988175	ALDH7A1 and PHAX	[32]
rs1476570	6	29809860	HLA-G	[32]
rs3830041	6	32191339	NOTCH4	[32]
rs6584283	10	101290301	Near NKX2-3	[32]
rs77969132	12	31594813	DENND5B	[32]
rs2730985	12	43130624	Near PRICKLE1	[32]
rs1886450	13	73986628	KLF5 and KLF12	[32]
rs4341754	16	80039621	WWOX and MAF	[32]
rs1078643	17	10707241	Near PIRT	[32]
rs13831	20	57475191	GNAS	[32]

A review study of the CRC prevalence trend in Asia [37] finds that several CRC case studies in Asian populations, especially South Korea, suggested a relatively lower rate mutation in CRC-related genes such as APC, K-ras, and p53 commonly found in Caucasian populations [38, 39]. A GWAS study in 2020 done by Asia Colorectal Cancer Consortium using data from 14 East Asian studies has identified 14 novel risk loci, 8 of were not replicated in populations with European descent [32]. Genes located in those loci are found to be related to colorectal tumorigenesis pathways such as Wnt signaling to beta-catenin and prostaglandin E2 catabolism. The study also finds that 11.7% of the familial risk of CRC in East Asian population may be attributed by these new variants combined with the common variants.

### Diet, Lifestyle, and Reproductive Factors

A number of lifestyle factors for colorectal cancer have been consistently observed across studies, although for the most part, these studies have been conducted in Western countries [40]. Systematic reviews of colorectal cancer in Asians have also confirmed several dietary factors including red meats, processed meats, preserved foods, saturated/animal fats, cholesterol, high sugar foods, spicy foods, tubers, or refined carbohydrates as risk factors for colorectal cancer [41]. The protective effects of calcium/dairy foods, vitamin D, general vegetable/fruit/fiber consumption, cruciferous vegetables, soybean/soy products, selenium, vitamins C, E, and B12, lycopene, alpha-carotene, beta-carotene, folic acid, and many other vitamins and minerals on the risk of colorectal cancer appears to be more inconsistent across studies, depending on the study design and population [41]. Aside from diet, physical activity and obesity are two important factors influencing the risk of disease [42]; both trends are also changing in Asian countries [43]. In a meta-analysis of 16 cohort studies, the most physically active individuals report a 23%–24% lower risk of colon cancer than the least active cohort; no association was observed for rectal cancers [44]. Between 1990 and 2013, there is an increase in the occurrence of obesity in the Asia-Pacific population, with a percentage change of 18.3%. The greatest prevalence of obesity is observed in the East Asian and Southeast Asian populations, which experienced an increase of more than 30% during that period [45]. In general, prevalence rates of obesity are still somewhat lower compared to western populations, but Asians have a higher percentage of body fat and visceral adiposity for the same body-mass-index (BMI) compared to those of Western origin [46]. Indeed, several meta-analyses among Asians have observed an increased risk for colorectal adenomas, precursor lesions, and colorectal cancer, as well as higher BMI and waist circumference [47, 48]. Unsurprisingly, diabetes mellitus has also been associated with an increased risk for colorectal cancer in several studies [49].

Based on biological studies, the relationship between obesity and colorectal cancer (CRC) explores the potential underlying mechanisms that connect the two. These two relationships in different aspects, such as nutriology, adipokines and hormones, inflammation, gut microbiota, and bile acids, provide evidence to support their role in CRC development [50]. Obesity-induced gut microbiota dysbiosis may lead to CRC tumorigenesis, while bile acids, particularly DCA and T-β-MCA, promote CRC progression [51, 52]. In addition, the author suggests that the elevated levels of insulin, IGFs, leptin, and inflammatory cytokines, and decreased levels of adiponectin in obese individuals may also contribute to CRC formation and development [53, 54].

In developed countries, a marginally elevated risk for colorectal cancer has been observed for alcohol consumers compared to non-alcohol consumers while cigarette smoking has been observed to increase the risk of colorectal cancers [55], particularly for right microsatellite unstable colon cancers in women [56]. Much of Asia has become high consumers of tobacco [57], although the association between smoking and colorectal cancer in Asians is less clear; a positive association is observed among the Chinese-descent populations of the relatively more developed Hong Kong and Singapore [58, 59], but not among the Shanghai Chinese population [60].

The role of reproductive factors and exogenous female hormone use have been extensively investigated in western populations, but less so in Asians. The Women’s Health Initiative Clinical Trial reports that postmenopausal women undergoing hormone replacement therapy shows a 40% reduction in colorectal cancer risk [61]. However, hormonal replacement therapy appears to have a detrimental effect on colorectal cancer risk after tumors have developed [61]. Results from the Japan Public Health Center-based Prospective Study on 48,511 women observe no association with hormone use; however, the findings highlight that late age at first birth is associated with a reduced risk of colon cancer in postmenopausal women [62]. Differences in study populations and potential heterogeneity by subsite (colon v. rectum), and other patient and lifestyle factors merit further investigations.

### Screening and Early Detection

Colorectal cancer has been described as an ideal disease for screening given the population-based estimates of prevalence, presence of early precursor lesions, and the noted effectiveness of various fecal and structural visualization methods of the colorectum. Overall, screening has been shown to decrease mortality from CRC through early detection and removal of precursor lesions such as adenomas and serrated lesions [63–65]; in particular, for polypectomy of low-risk versus high-risk adenomas [66]. The two main forms of screening are [1] fecal occult blood tests, which detect blood in the stool such as the guaiac FOBT (gFOBT) and the fecal immunochemical (or immunohistochemical) test (FIT); and [2] structural tests, sigmoidoscopy or colonoscopy, which are used to visualize the interior of the colorectum using a scope. The U.S. Preventive Services Task Force recommends screening for colorectal cancer using fecal occult blood testing, sigmoidoscopy, or colonoscopy for adults beginning at age 50 years and continuing until age 75 years [67]. In 2004, in light of the absence of national and regional guidelines on prevention and screening, the Asia-Pacific Working Group on Colorectal Cancer was formed. The group’s publication in 2008 [12], endorses the screening for colorectal cancer beginning at age 50, as is recommended in several other developed countries. A review of CRC screening in Asia indicates that the likelihood of finding CRC-related neoplastic lesions triples in patients aged over 50 year old. This significant increase in diagnostic likelihood for CRC informs the consensus in Asia and in other parts of the world to begin screening at age of 40 [68]. Similar to recommendations by the U.S. Preventive Services Task Force, fecal occult blood tests (FOBT, guaiac-based and immunochemical tests), flex sigmoidoscopy, and colonoscopy are recommended methods of effective screening. In resource-limited countries, FOBT is recommended as the first choice for CRC screening with any polyps 5–9 mm in diameter removed via a subsequent endoscopic exam. Following a negative colonoscopy, it is recommended that a repeat examination should be performed in 10 years. Given the rising incidence of right-sided colon tumors in Asia [23, 69, 70], colonoscopy-based screening is critically important [68]. Apart from FOBT, colonoscopy, and sigmoidoscopy, an effective early screening method performed by countries with large populations such as China is to utilize an early detection questionnaires to recognize some CRC high-risk factors such as precancerous lesions [71]. Moreover, the mode of opportunistic screening in China can also be carried out in hospitals, community clinics, and medical centers in densely populated countries such as Indonesia, to anticipate the large amount of financial funding that costs beyond that of allocated by the national finance budget [71]. In addition, colonoscopy as a means of opportunistic screening is also popular in Japan, South Korean, and Taiwan but the use of sigmoidoscopy is less popular there [72].

Moreover, public health priorities must shift to address these CRC trends in Asian populations: 1) CRC screening in Asian countries should be made a national health priority, and 2) extensive studies are needed to determine barriers to CRC screening, such as public education regarding the importance of cancer prevention and the engagement of physicians with primary healthcare [73]. In addition, an in-depth evaluation of the public acceptance of a wide variety of screening modalities based on cultural factors is also essential [74]. which can contribute to determining the risk stratification in CRC screening along with the availability of public health resources [73].

One of the crucial efforts in early CRC detection and cancer control is related to the development of a cancer registry with the main objective of building a system to collect and organize data on all cancer cases in order to build statistical models, such as polygenic models, to determine the progression of cancer within a population [75–77]. The data is principally gathered periodically from various cancer institutions such as cancer hospitals. Cancer registries help clinicians to make personalized follow-up treatments for patients [78] and to act as a surveillance system for at-risk individuals [79]. Centralized database management integrated into a cancer registry can also provide a cancer epidemiological tool for researchers [80].

Principally, early detection is required to minimize the high rate of mortality among CRC patients due to the late start of cancer treatments [75]. This requires a great number of studies about the role of various lifestyle and genetic aspects toward the progression of CRC in Asian populations [43, 73, 81–83]. Moreover, the phenotypic and genotypic data from these studies can be used to build polygenic models to predict a patient’s risk score for CRC [81]. The inclusion of unique genetic profiling in CRC assessments provides both researchers and clinicians to develop more personalized treatment for the patients [84]. The Asia-Pacific Colorectal Screening Score based on age, gender, family history, and smoking stratified patients into moderate-risk (2.6-fold increased risk) and high-risk tiers (4.3-fold increased risk) compared to the average-risk tier. The classification method is an important starting point for further validation, offering resource-limited Asian countries the potential ability to focus on subgroups of the population with the highest risk who may benefit greatly from routine screening [85].

In high-income Asian countries like Japan, South Korean, Singapore, and Taiwan**,** elaborate cancer registries have been developed to accomodate data acquisition and management of various cancer-related data. For example, Korea Cancer Big Data Platform provides an framework to manage various types of cancer data (medical records, genomics data, blood and tissue data, etc) with a secure privacy system for ten types of cancer [86]. However, there is a huge gap of cancer registry development between the high-income countries and low-income countries in Asia. Apart from the technicality and capability issues, the main challenge of cancer registry developments in most low-income countries is to obtain reliable cancer statistics [87]. There are several collaborational efforts among several national cancer centers in Asian countries with the main goal to create a long-term framework for education and training about cancer prevention and control as well as an alignment/standardization on cancer registries. One of the most recent collaboration efforts is the Asian National Cancer Centers Alliance (ANCCA) with the active participating members are China, India, Indonesia, Japan, Korea, Mongolia, Singapore, Thailand, and Vietnam [88]. The collaboration had planned three phases of ANCAA from 2020–2031, including a protocol for Asian-specific medical standards for cancer registries.

## Summary

The trend of Asian colorectal cancer incidences reaches the comparable rate of cases in several countries of America, Australia, and Europe. Many studies about genetic susceptibility to colorectal cancer have found several specific variants in Asian populations. Rapid changes in lifestyle and dietary behavior are associated with an increased risk of Asian colorectal cancer trends in addition to genetic factors. Due to the lack of proper health facilities and infrastructure, most low-resource nations in Asia employ inefficient procedures for the early diagnosis of precancerous lesions and prevention of colorectal cancer [89, 90]. Across the Asia Pacific region, multiple studies have explored the challenges of CRC screening within diverse cultural and sociopolitical settings. Key barriers identified in these studies include insufficient awareness of CRC screening and test characteristics, inadequate financial support, and a lack of health insurance coverage. However, several developed countries in Asia, such as China and Japan, are running population-based screening programs for CRC in the age range of 40–70 years. In Europe and North America, routine examinations through screening protocols on the elderly (over 50 years) have led to a decrease in the incidence of colorectal cancer [91, 92]. The effect of screening on colorectal cancer risk has been extensively studied in other countries [93]. This breadth of information is still insufficient to understand the current increase in colorectal cases. Therefore, an in-depth and comprehensive study is needed for the diverse ethnic/regional populations in the Asian continent.

Despite our goal of identifying the comprehensive trend of CRC-related topics in several Asian countries, the data that can be retrieved from the CI5 website (Cancer Incidence in Five Continents) published by the International Agency for Cancer Research (IARC) used in this review is limited largely to the time period from 1999 to 2010 for available continuous and complete data at the national level that encompasses both genders and a wide age range. In addition, the CI5 database was also discontinued for several countries, especially countries from West and South Asia countries, which is one of the primary reasons that this review focused on countries that had continuous data up to 2010, namely, those found within East and South East Asia. Additionally, the analyses performed were based on geographical divisions. However, the regions analyzed contain a wide breadth of diversity based on ethnicities and cultures. Therefore, our analyses do not include how the diversity of cultures and ethnicities contributes to the changes in CRC trends in the countries described. Therefore, a further in-depth and comprehensive study will be needed to account for these factors in order to provide a more accurate picture of the changes of CRC rates within these parts of Asia.
